# Maternal emotion socialization in Chinese, Indian, and European American families: socialization goals as a culturally embedded factor

**DOI:** 10.3389/fpsyg.2026.1772882

**Published:** 2026-04-23

**Authors:** Meingold Hiu-ming Chan, Tripti Kathuria, Wolfgang Friedlmeier, Xin Feng, Shagufa Kapadia

**Affiliations:** 1 Department of Human Development and Family Science, Purdue University, West Lafayette, IN, United States; 2 Department of Human Development and Family Studies, The Maharaja Sayajirao University of Baroda, Vadodara, India; 3 Psychology Department, Grand Valley State University, Allendale, MI, United States; 4 Department of Human Sciences, The Ohio State University, Columbus, OH, United States

**Keywords:** cultural variations, culturally embedded factors, emotion socialization, socialization goals, within-culture differences, multi-group structural equation modeling

## Abstract

**Introduction:**

A growing body of research has documented cultural variations in family emotion socialization (ES) that teach children about their experience and expression of emotions. Theoretical models have been proposed to elucidate the origins of these differences, suggesting that emotion-related socialization goals could be a key culturally embedded factor that shape ES across cultures.

**Methods:**

The current study integrated two cross-cultural studies (n = 193, mean age = 3.37 years, range = 1.5–5.96 years) involving four cultural groups—Hong Kong Chinese, Beijing Chinese, Indian, and European American—to address the universal and cultural-specific process involved in the emergence of cultural variations in maternal ES across and within-culture.

**Results:**

First, we demonstrated that not only there were cross-cultural differences between European American and Asian mothers, but also similarities and differences in ES practices across Asian groups (Indian vs. Chinese) as well as within Chinese cultures. For example, Chinese mothers reported higher endorsement of training responses than European American mothers, and Beijing Chinese and Indian mothers endorsed higher levels of problem-focused responses. Second, we showed that emotion-related socialization goals served as a culturally embedded factor that contributed to ES practices. Specifically, relational emotion competence goals were linked to more distress responses, minimization, and training, while individualistic emotion competence goals were related to less distress and more emotion-focused responses. However, importantly, multi-group SEM models revealed that these associations were culturally specific.

**Discussion:**

The strength and challenges of harmonizing independently conducted cross-cultural studies were discussed.

## Introduction

Family emotion socialization (ES) is a primary pathway for children to develop their understanding of emotions and competence in expressing and regulating emotions (Eisenberg et al., 1998; Friedlmeier et al., 2011; Morris et al., 2007). Children’s ability to understand and regulate emotions in a socially-appropriate manner are key developmental milestones and robust predictors of later psychopathology, social relationship, and academic achievement (Aldao et al., 2016; Denham et al., 2015; Eisenberg, 2020). Although the process of family ES occurs in most cultures, the specific practices can be culturally delineated. An emerging literature has begun to document cross-cultural variations in caregivers’ ES practices (Raval and Walker, 2019; Yeo et al., 2022). Nonetheless, this area of research still has major limitations as much of the research on parental ES is conducted in Euro-American contexts and research on ES in Asian cultures have only recently begun to “emerge on the sidelines” (Raval, 2023, p. 142). In a recent review on ES, only about half of the 31 reviewed studies included Asian families and even fewer compared across Asian groups (Raval and Walker, 2019). Families from diverse Asian cultures are often assumed to reflect similar cultural values or norms and reduced to broad concepts such as collectivism (Raval, 2023). Thus, the heterogeneity of Asian cultures has not been fully appreciated, both across countries in Asia (e.g., India, China) as well as within-country (e.g., Beijing Chinese vs. Hong Kong Chinese). Yet, the intersections of socioeconomic status, sociopolitical contexts, and individuals’ beliefs and experience could result in nuanced variations across and within-Asian cultural groups (Wang, 2023). A recent conceptual model on caregivers’ ES in culturally diverse groups has proposed the importance of studying culturally embedded factors that may underlie these cross- and within-cultural differences, such as caregivers’ socialization goals related to child’s emotional development (Raval and Walker, 2019). Thus, the current study addressed these gaps by drawing on data from three Asian cultural groups—Hong Kong Chinese, Beijing Chinese, and urban Indian—and European American (EA) in the Midwest US. These cultural groups likely reflect diverse cultural models along the spectrum of interdependence-independence. We investigated the universal and culture-specific process of how caregivers’ emotion-related socialization goals, specifically emotion competence (EC) goals, contribute to their ES practices in different cultural contexts among families of 2 to 5-year-old children.

### Culturally informed research on caregivers’ ES practices

The importance of a cross-cultural perspective on caregivers’ ES has been well-recognized (Friedlmeier et al., 2011) and incorporated in major theoretical frameworks of the field, such as the Heuristic model (Eisenberg, 2020; Eisenberg et al., 1998) and the Tripartite model (Morris et al., 2007). While the role of culture was recognized but considered beyond the scope of their review in the Tripartite model (Morris et al., 2007), the Heuristic model included cultural factors, such as emotion-related beliefs, norms, and values, as potential predictors of ES practices (Eisenberg, 2020; Eisenberg et al., 1998). The focus on the role of culture is further expanded upon in the more recent Integrative model that coined the term “culturally-embedded factors” and called for more cross-cultural studies on ES (Raval and Walker, 2019). Guided by these models, a growing number of culturally informed ES studies have included more diverse samples from around the world, including but not limited to, China, India, Indonesia, Nepal, South Korea, and Turkey, to combat the overrepresentation of White, Euro-American samples (Corapci et al., 2012; Raval, 2023; Raval and Walker, 2019; Röttger-Rössler et al., 2013; Song et al., 2023). The current study built on these models and extended the existing literature by directly examining how culturally embedded factors contribute to cultural variations in ES in four cultural groups.

Numerous pathways of family ES have been identified in theoretical models, including emotion-related parenting practices (e.g., responses to children’s emotions, emotion discussion in family), observation or modelling, and emotional climate of the family (Morris et al., 2007). Most relevant to the current study is parental responses to children’s emotions. Most studies on ES practices conducted inside and outside of the U.S. focused primarily on children’s negative emotions, given its direct relevance to emotion dysregulation and later psychopathology, although the importance of socialization of positive emotions have been increasingly recognized (Breaux et al., 2022). Existing studies have primarily adopted the Coping with Children’s Negative Emotions Scale (CCNES) developed based on research and theories grounded in the U.S. cultural contexts (Fabes et al., 1990; Friedlmeier et al., 2011). Six types of parental responses to children’s negative emotions were included in CCNES: (1) *Expressive encouragement* refers to parents validating and encouraging their children to express and discuss emotions; (2) *Emotion-* and (3) *problem-focused* responses refer to providing comfort or other ways to soothe children’s emotions and helping children to resolve the problems that induced emotions; (4) *Minimization* involves invalidating and restricting children’s emotions or the reasons of the emotions, while (5) *punitive* responses are harsh or critical behaviors such as punishing or yelling at the child. Lastly, parents may become (6) *distressed* themselves in the face of their children’s negative emotions. The first three responses were labelled as supportive responses in US-based studies, while the latter three responses were typically labelled as ‘non-supportive’ responses in US-based studies (Friedlmeier et al., 2011).

Some studies expanded categories of parental responses in CCNES by adding strategies grounded in other cultural contexts. For example, Chan et al. (2009a,b) added *reflection-enhancing* that emphasized children’s self-reflection on the causes and consequences of their own emotions and parental *training* responses that focuses on teaching their children about emotion-related rules and justifying with moral reasons based on Hong Kong Chinese families with school-aged children. Focusing on these more culturally salient parenting practices, studies found that Chinese mothers of school-aged children used more supportive strategies than Italian mothers (Chan et al., 2009b; Fiorilli et al., 2015). Relatedly, Chinese mothers of 2- to 5-year-olds were more likely than EA mothers to respond with training, discussion, and education strategies towards child’s aggression or withdrawal from peers (Cheah and Rubin, 2004). Similarly, a study in India on 5 to 9-year-old children developed culturally salient hypothetical vignettes similar to CCNES and added a new response—*(un)acceptability of emotion expression—*and distinguished problem-focused responses to solution vs. explanation-focused (Raval and Martini, 2009), which were later used in a series of Indian-based studies (Raval et al., 2013, 2014, 2016; Raval and Martini, 2009). These findings showed that it is important to expand the Euro-American focused measure of ES practices to include more culturally salient behaviors when examining ES in other cultural groups.

### Cross- and within-cultural variations in caregivers’ ES practices

Cultural differences in ES practices between EA in the U.S. and Asian groups, specifically Indian and Chinese, can be derived from single culture studies and has also become evident in recent cross-cultural studies. In the U.S., EA caregivers tended to report using supportive ES responses more often than non-supportive responses (Friedlmeier et al., 2011). In contrast, Chinese mothers of first graders reported the frequency of using both minimization and expressive encouragement to a similar extent (Chan et al., 2009b; Tao et al., 2010). Indian mothers of school-aged children generally rated minimization higher than expressive encouragement (Raval and Martini, 2009). From cross-cultural studies, Indian mothers of school-aged children were found to report more culturally salient ES practices such as explanation-oriented responses than White U.S. mothers and fewer problem-focused responses as measured by CCNES (Raval et al., 2013). In contrast, U.S. mothers reported more expressive encouragement assessed by CCNES than Indian mothers (Raval et al., 2013). Similarly, Indian immigrant mothers of school-aged children in the U.S. reported using more non-supportive responses, including magnify, punish, neglect, and override responses, than White American mothers (Mccord and Raval, 2016).

Limited cross-cultural research between U.S. and Chinese mothers’ ES focusing on responses to children’s emotions was conducted (Raval and Walker, 2019). However, ES studies focusing on discussion of emotions indicated cross-cultural differences. For example, Chinese mothers of 3-year-olds were more likely than their U.S. counterparts to refer to children’s behaviors (Doan and Wang, 2010) and teach children about the moral appropriateness of their emotion expression or behaviors (Wang, 2001). Using a subset of the cross-cultural data in the current study, our team published a recent qualitative study on Hong Kong Chinese, Beijing Chinese, and EA mothers’ dismissive responses to children that added to this observation (Chan et al., 2026). Specifically, we found a training philosophy of ES that focused on instilling moral values, teaching life lessons, and training children to follow social rules for emotion expressions, which was more salient in Chinese mothers than U.S. mothers. Therefore, growing evidence has supported that ES practices varied across cultural contexts. However, what remains underexplored is the variations across Asian groups or within culture.

There is a scarcity of research that has examined cultural differences across Asian groups. The majority of within-culture ES studies were conducted in the U.S., comparing mothers from different ethnic backgrounds and have found shared and distinct patterns of ES (Brown et al., 2015; Mccord and Raval, 2016; Nelson et al., 2012; Parker et al., 2012; Raval and Walker, 2019). Given the underappreciation of heterogeneity across Asian cultures, very few studies were conducted to compare across Asian groups (Cole et al., 2006; Ersay, 2014; Raval et al., 2013; Raval and Martini, 2009). Parenting and socialization in urban families in China and India are likely to share similarities due to their overall emphasis on familial interdependence and social harmony yet some distinctions may emerge due to their differing belief systems of Confucian and Hindu values (Rao et al., 2003). For example, in the traditional Hindu view, young children are considered divine, born with innate disposition and adult-child interactions are largely guided by indulgence rather than training and socialization (Saraswathi and Ganapathy, 2002). In contrast, Confucian beliefs focused on how to overcome children’s innate tendencies by shaping their environment and that include parenting effort and training. The handful of studies that examined Indian and/or Chinese families showed both similarities and differences (Trevethan et al., 2021; Yeo et al., 2022). On the one hand, mothers of adolescents from both cultures were found to have similar profiles of ES practices in a cross-cultural study (Trevethan et al., 2021) and culturally salient behaviors of similar nature when socializing school-aged children’s emotions (e.g., reflection-enhancing in Chinese context and “making the child understand” or not talking to the child briefly, in Indian context) has been reported (Chan et al., 2009b; Raval and Martini, 2011). On the other hand, Indian, Chinese, and Singaporean parents reported different levels of endorsement of ES responses to adolescence’s emotions (Yeo et al., 2022). Therefore, it is crucial to illuminate the nuanced heterogeneity in ES across Asian cultures.

Within-cultural differences of ES behaviors in China are underexplored. However, interactive effects of historical (e.g., colonization), societal (e.g., modernization), cultural (e.g., socialization goals), demographic (e.g., socioeconomic status), and individual (e.g., beliefs about emotions) factors may contribute to within-cultural variations in Chinese parenting practices, including ES practices (Chan et al., 2026; Wang, 2023). This study focuses on comparing Hong Kong, a former colony of Britain and currently a special administrative region of China, and Beijing, the capital of China. Although Hong Kong has been described as a “melting pot” of Eastern and Western values (Chan and So, 2016) given its 155-year history of British colonization and assumed to be more westernized than other parts of China, recent research has presented opposing evidence. Hong Kong Chinese parents reported higher level of parental control, lower level of warmth, and allowed less autonomy in their children, compared to their mainland Chinese counterparts (Fung et al., 2017). Some possible reasons for these findings include the recent, rapid industrialization and social change in mainland China as compared to the relatively stable Chinese society in Hong Kong since colonization, as well as the one-child policy implemented in mainland China that may explain the higher child-directed parenting style in mainland China compared to Hong Kong (Fung et al., 2017).

To our knowledge, no studies to date have directly compared ES practices between Hong Kong and mainland Chinese parents. The qualitative portion of our project revealed interesting within-cultural differences between Hong Kong and Beijing Chinese mothers’ meta-emotion philosophy; specifically, Hong Kong mothers had a stronger concern of social judgement and were more often discussing their inability to change their dismissive ES behaviors than Beijing mothers (Chan et al., 2026). In independent studies, Beijing Chinese mothers endorsed problem-focused and emotion-focused responses more often than other ES practices as assessed with CCNES (Tao et al., 2010). As aforementioned, Hong Kong mothers reported using training and reflection-enhancing responses more often than all the other responses included in CCNES (Chan et al., 2009b; Chan, 2012). Direct comparison between Hong Kong and Beijing Chinese using the same measure that include these culturally salient responses could be informative for whether the within-cultural parenting observed in past studies also extended to ES practices. Furthermore, in conjunction with the efforts to illuminate across and within cultural variations of ES practices, research has now directed more attention on to unravelling the mechanisms underlying the emergence of these cultural variations.

### Culturally embedded factors driving ES: Emotion competence socialization goals

Guided by the conceptual framework for culture and ES that extended Keller’s cultural model of independence and interdependence (Keller et al., 2006; Raval and Walker, 2019), we focuses on culturally embedded factors that guide ES to help explain cultural variations in ES. Three culturally embedded factors were proposed by Raval and Walker (2019), including caregivers’ socialization goals, preferences for communication, and beliefs about emotion. The current study focuses on socialization goals, specifically emotion-related socialization goals, referred to as emotion competence (EC) socialization goals from here onwards. Parents across cultures share a similar goal—to raise and socialize their children to become competent members of their community based on the shared beliefs and values in their society (LeVine, 1974). Ample research has documented cultural variation in socialization goals (or beliefs) across EA and Asian mothers (Chen et al., 2015; Raval and Walker, 2019). While EA mothers focused on promoting children’s autonomy and intrinsic motivation, Indian and Chinese mothers focused on duty to one’s family, filial piety, and academic achievement (Chao, 2000; Trommsdorff et al., 2012). These socialization goals serve as an important foundation for parenting practices (Rao et al., 2003) and have begun to be recognized as a potential driving factors of parental ES practices (Raval et al., 2014; Raval and Walker, 2019). Specifically, parents’ EC socialization goals referred to the goals of fostering their children to develop the set of EC that are culturally valued in their local communities (Chan, 2011). EC that is considered adaptive varies across cultures, depending on the demands of the social environments, the socioeconomic structures of a society, as well as its historical background (Keller et al., 2006; Tamis-LeMonda et al., 2008); hence, EC socialization goals of parents also vary across cultures.

EA cultures are typically assumed to have an independent cultural model that prioritizes individualistic values including autonomy, separateness, and attainment of personal goals (Markus and Kitayama, 1991). Thus, understanding of one’s own and open expressions of emotions is greatly valued within this cultural model. Not only positive emotions are encouraged to be expressed, negative emotion expressions are also accepted (Garrett-Peters and Fox, 2007). Given these values, EA parents may endorse an *individualistic* EC goal—to promote children’s ability to understand and express their emotions, especially ego-focused emotions such as pride and anger (Friedlmeier et al., 2011; Tamis-LeMonda et al., 2002), so that their feelings could be acknowledged, and their desires met.

In contrast, Asian cultures are generally assumed to have an interdependence orientation that values the interrelationship between individuals and maintenance of social harmony. Hence, people in this cultural model tend to take others’ feelings into consideration or read others’ “faces” when expressing one’s emotions and are encouraged to control their emotions to maintain social harmony (Chan et al., 2009b; Wang, 2006). Both traditional Chinese medicine and the teaching of Taoism believe that strong emotions are harmful to one’s health and body (Cheah and Rubin, 2003; Garrett-Peters and Fox, 2007), which could drive Chinese parents to be more eager to socialize self-control or suppression of emotions in their children. Similarly, in Hindu tradition, the ultimate goal of human life is to attain *moksha* (spiritual salvation) by adhering to strict guidelines for appropriate conduct (Pandit, 2001), including the regulation and dismissal of inappropriate behaviors to maintain social harmony. Consequently, Indian caregivers often encourage children to control or dismiss “uncivilized emotions” (Menon, 2000, p. 47) from an early age (Kathuria et al., 2023). Interdependent cultures, like Chinese and Indian cultures, value *relational* EC goals, which includes understanding emotion display rules and consequences of emotions to interpersonal relationship. Indeed, empirical evidence suggested that Hong Kong Chinese mothers value promoting relational more than individualistic EC goals in their children (Chan, 2011). A recent study showed Indian mothers of toddlers emphasizing balancing collectivistic goals with individual autonomy (Kathuria et al., 2023). Although there is a scarcity of research that examined differences in EC goals within Asian cultures in childhood, heterogeneity across Asian groups were found in a recent study among parents of adolescents; specifically, Indian, Chinese, and Singaporean parents reported different levels of independence/interdependence self-construal as well as ES responses to adolescents’ emotions (Yeo et al., 2022). Thus, parental EC goals could drive their ES practices, leading to both across- and within-cultural variations of ES practices. However, within-Asian cultural differences among parents of younger children are still underexplored—one of the gaps that the current study aims at filling.

The associations between EC socialization goals and ES practices have been primarily investigated in studies within Asian context. For example, in Hong Kong Chinese families, individualistic EC goals were linked to more emotion encouraging responses, while relational EC goals showed a negative association (Chan et al., 2009b). Similarly, relational EC goals were also linked to Indian mother’s explanation-oriented ES behaviors (Raval et al., 2014). However, it is unclear whether EC socialization goals are also a relevant factor in EAs and how cultural contexts may moderate these associations. Among the limited studies that examined the effect of socialization goals on child rearing across Asian groups, mixed findings were reported. While one study has shown no moderating effect of cultural groups when examining the association between mother’s relational EC goals and ES practices in India and China (Raval et al., 2018), past studies have shown that maternal socialization goals (filial piety vs. socioemotional development) were differentially associated with child-rearing practices in Chinese and Indian mothers, attributing the cultural values of Confucian and Hindu beliefs, respectively (Rao et al., 2003). Similarly, within-cultural differences in the associations between socialization goals and ES responses were found across rural and urban Chinese communities, with relational EC goals linked to ES practices in rural, but not urban, communities (Wu et al., 2025). Hence, more empirical research is still needed to unravel the universal and culturally specific process of EC socialization goals as culturally embedded factors that drive cultural variation of ES behaviors.

### The present study

Cross-cultural comparisons of maternal ES practices between EA and Asian families are primarily conducted with parents of school-aged and adolescent children, while studies on parents with preschoolers are scarce in the literature. Maternal ES is especially important during the early childhood period since children are still heavily dependent on other-regulation of their emotions, often by their mothers, before developing ability to self-regulate (Holodynski and Friedlmeier, 2005; Sameroff, 2010). Moreover, maternal ES practices can change based on their developmental expectations (Chan et al., 2009a) and their response to their children’s developmental level (Eisenberg, 2020). Therefore, it is key to expand our understanding on maternal ES of preschoolers and toddlers. There are even fewer studies in this developmental period that shed light on the within-cultural variations across Asian groups. Our understanding of how these cross- and within-cultural variations emerge and are shaped by culturally embedded factors is limited.

The current study addresses these gaps by leveraging two existing datasets representing four cultural groups—China (Hong Kong and Beijing), urban India, and EA in the Midwest US, likely reflecting diverse cultural models along the spectrum of interdependence-independence—and using culturally informed measures to examine maternal ES practices of children aged 2 to 5. Our study had three aims. First, we aim to document and compare the cultural norms of maternal ES practices and EC goals in the four cultural groups. We hypothesize that Asian mothers would report using more minimizing and training responses than EA mothers (H1a). We expect some level of variations across the Asian cultural groups, specifically with Hong Kong mothers reporting more use of unsupportive ES responses (e.g., minimization) than other Asian groups, as informed by the qualitative portion of this study (Chan et al., 2026) (H1b). Also, we hypothesize that the three Asian cultural groups would report higher relational EC goals than EA mothers, while EA mothers would endorse higher individualistic EC goals (H1c).

Our second aim is to investigate how culturally embedded factors—specifically, EC goals—are associated with maternal emotion socialization practices. We hypothesize that relational EC goals would be associated with higher use of minimizing and training responses, while individualistic EC goals would be associated with higher use of supportive responses (e.g., problem-focused and emotion-focused responses) (H2). Third, to examine universal and culture-specific processes, we explore how these associations may be moderated by cultural groups using a multi-group path model.

## Materials and methods

### Participants

Data in the current study were drawn from two existing cross-cultural studies conducted in the U.S. and China, as well as the U.S. and India. A total of 193 children (91 females, mean age = 3.37 years, range = 1.5–5.96 years, SD *=* 1.33 years) and their mothers in urban India (*n* = 50), China (Hong Kong and Beijing) (*n* = 62), and the Midwest U.S. (*n* = 80; *n* = 31 in Ohio and *n* = 51 in Michigan) were included in the sample. Participants were recruited through local school districts, day care centers/preschools, community centers, social media (e.g., Facebook, WeChat), and online advertisements in Hong Kong, Beijing, and U.S. Participants in India were recruited through playgroups, daycare centers, and through personal contacts. The language spoken at home was English in the U.S., Chinese (Cantonese in Hong Kong and Mandarin in Beijing), and Gujarati/Hindi/English in India. The demographic information of all participants is presented in Table 1.

**Table 1:** Participant characteristics.

Demographic variables	EA in Ohio (*n* = 31)	EA in Michigan (*n* = 51)	Hong Kong Chinese (*n* = 32)	Beijing Chinese (*n* = 30)	Indian (*n* = 50)
Child age (years)	4.36 (0.22)	2.14 (0.30)	4.93 (0.61)	4.77 (0.61)	2.22 (0.36)
Child sex (*n* of Male)	15 (48.4%)	26 (51.0%)	16 (48.5%)	19 (57.6%)	28 (56.0%)
Mother education	Less than grade school (*n* = 1) High school/GED (*n* = 1) Junior college/associate degree (*n* = 1) Some college/specialized training (*n* = 1) Bachelor’s degree (*n* = 17) Postgraduate (*n* = 10)	Secondary school (*n* = 3) Some higher education incomplete (*n* = 9) Bachelor’s degree (*n* = 17) Postgraduate (*n* = 21)	Form 4–7 (*n* = 4) High school diploma (*n* = 6) Associate degree (*n* = 3) Bachelor (*n* = 15) Postgraduate (*n* = 4)	Associate degree (*n* = 1) Bachelor (*n* = 24) Postgraduate (*n* = 7)	Secondary (*n* = 8) Graduation (*n* = 24) Postgraduate (*n* = 18)
Marital status	Married (*n* = 29) Living with someone (*n* = 1)	Married (*n* = 46) Single (*n* = 5)	Married (*n* = 32)	Married (*n* = 30)	Married (*n* = 50)

### Procedures

The cross-cultural study in the U.S. (Ohio) and China (Study A) was approved by the Institutional Review Board (IRB) of the Ohio State University, while the study in the U.S. (Michigan) and India (Study B) was approved by the IRB of Grand Valley State University. The co-authors at each site completed a site-specific local context worksheet to ensure that the study procedure also aligned with the unique local regulations. We obtained informed consents from all participants using a consent form in their native language (i.e., Chinese in Hong Kong and Beijing; English in the U.S.; Gujarati/English/Hindi in India, depending on the preference of the participants).

In study A, all mother–child dyads participated in a 1.5 to 2-hour home or lab visit. Mothers completed questionnaires about their demographic information, ES practices, and child outcomes. Children completed a series of child assessment for emotional and cognitive outcomes, which was not used in the current study. All interviews and assessments were translated and administered in the participants’ native languages. Translation from English to Chinese was done by the first author, who is fluent in both English and Chinese and was born and raised in Hong Kong, and bilingual research assistants who had training in child development. The translation for the Hong Kong site was then reviewed and revised by a certified bilingual translator in Hong Kong. The translation for the Beijing site was reviewed by the Beijing research team, who are more familiar with the cultural context of Beijing. Mothers in this sample were relatively highly educated, with the majority having at least a bachelor’s degree in all three sites (60% in Hong Kong, 90% in Beijing, and 70% in the U.S.) (Table 1).

In Study B, all mother-toddler dyads participated in approximately 1.5–2 hour in a research lab. Mothers participated in semi-structured interviews on ES practices and completed questionnaires on socio-demographic information and socialization goals. All the tools were translated and used in participants’ native/preferred language (i.e., Hindi or Gujarati, and in few cases English). The tools were translated from English to Hindi by the second author, who is well versed with both Hindi and English and was born in India. Translation from English to Gujarati was done by a research assistant whose native language was Gujarati and who was fluent in both English and Gujarati.

### Measures

#### Maternal ES practices

Survey measures were used in Study A conducted in China and Ohio, U.S. and interview were used in Study B conducted in India and Michigan, U.S. In China and Ohio, U.S., maternal ES practices, specifically reactions to children’s negative emotions, were measured with the well-established Coping with Child Negative Emotion Scale (CCNES; Fabes et al., 1990) and an additional survey that included more culturally salient reactions of Chinese parents—training response (Chan et al., 2009a, b). Together, seven ES behaviors were assessed, including distress, punitive, expressive encouragement, emotion-focused, problem-focused, minimization, and training responses.

The CCNES is commonly used to measure mothers’ perceived reaction to their child’s negative affect in distressing situations (Fabes et al., 1990). The questionnaire provided 12 hypothetical scenarios in which children exhibit negative emotions. These scenarios are common emotionally eliciting situations that young children are exposed to, for example, showing disappointment when received a gift they did not like. The CCNES contains 6 subscales of parents’ typical responses: distress (experiencing emotional distress such as feeling angry or upset), punitive (using punishment or any harsh/critical behaviors), expression-encouraging (encouraging their child to express emotions such as asking why he/she was sad), emotion-focused (comforting their child or focus on helping them feel better), problem-focused (helping their child to problem solve), and minimizing reactions (telling their child it’s no big deal or they are overreacting). Mothers reported their likelihood of responding to their children’s negative emotions with each of the six possible ways on a 7-point Likert scale with 1 as “*very unlikely*” and 7 as “*very likely*” and received a score on each subscale. Additionally, the training subscale, a more culturally salient response for Chinese parents, from the 12-item Mother’s Response to Child Emotion Scale (MRCES) were also used (Fiorilli et al., 2015). Training involves directly telling the child that their reactions are inappropriate and justify with social rules/moral reasons. Mothers reported their likelihood of responding to their children’s negative emotions on a 6-point Likert scale with 1 as “*very unlikely*” and 6 as “*very likely*.” The internal consistencies of all subscales except training response were good in all three sites (US: *α* = 0.77–0.86, HK: *α* = 0.83–0.91, Beijing: *α* = 0.75–0.93). The reliability of the training subscales was still low after removing two items that had low correlations with other items (6F “Tell my child that he/she should try to play. As other children are playing happily, it is not so scary.” and 12F “Tell my child: even though he/she wins, he/she should not brag. It is too arrogant and no one can win every time.”), U.S. (*α* = 0.63), Hong Kong (*α* = 0.73), Beijing (*α* = 0.51). However, given the construct validity and salience of this response in Chinese culture (Chan, 2012), we decided to keep this subscale in our analysis. Raw scores for ES practices before standardized are in Supplementary Table 1A.

In India and Michigan, U.S., an innovative methodology—a semi-structured interview using vignette designed based on CCNES (Corapci et al., 2018) (Supplementary material)—was used to obtain open-ended responses of maternal reactions to negative emotions of children, which were later coded. Mothers were presented with the vignettes that specified the emotion eliciting situation and toddlers’ emotions developed from a prior pilot study (Corapci et al., 2018). The mothers were asked to remember or imagine each situation with their child who is participant in the study and report how they would respond in such situation. Among the 10 scenarios, there were four scenarios (1, 4, 5, and 9)—two vignettes for sadness and two for fear—that were adapted from CCNES and the responses for these scenarios were used for this study. An example of the vignette for sadness is where a toddler participated in some group activities and made a mistake and looked sad, while one for fear is children getting an injection and separation from mother. The coded ES behaviors that aligned with five of the seven aforementioned behaviors—distress, emotion-focused, problem-focused, minimization, and training—were used in our analyses.

While the interview allowed for more open-ended responses and the opportunity to explore culturally salient responses that go beyond the categories in existing surveys, we were unable to capture those responses in this study when we harmonized the interview data with our survey data with pre-existing scales. We harmonized these data to the best of our ability by creating proportions (i.e. the frequency of specific code practice out of total number of coded practices) for our interview measure then standardizing (z-score) our variables across all groups (mean = 0, SD = 1 for each variable). The standardized variables were used in all analyses, including multivariate analysis of covariance (MANCOVA) and path models. Preliminary data exploration showed that despite using different measures for ES practices (survey in Ohio vs. interview in Michigan), the two US groups did not show statistically significant differences in endorsement for ES practices (all *p*s > 0.05 as shown by multiple regressions adjusting for child age, sex, and maternal education; see Supplementary Table 1B for details). This gives us some confidence that the two measures generated results that are comparable.

#### Maternal EC goals

We also measured mothers’ emotion-related socialization goals, specifically individualistic and relational EC goals, using the Parental Goals for Relational and Individualistic Emotional Competence Scale (Chan et al., 2009b). The scale includes two subscales with 10 items measuring individualistic EC goals (e.g., “My child can express his/her emotions and the reasons behind them in front of elders or authority figures”) and 10 items capturing relational EC goals (e.g., “My child can control his/her emotions so as to maintain interpersonal relationship”) on a 6-point Likert-type scale, from 1 (very unimportant) to 6 (very important). The internal consistencies for these scales were good in most sites, US: *α* = 0.83, HK: *α* = 0.85, Beijing: *α* = 0.84 for relational EC goals and US: *α* = 0.78, HK: *α* = 0.61, Beijing: *α* = 0.75 for individualistic EC goal, but low in India (*α* = 0.58 and 0.54, respectively). However, all item intercorrelations were higher than 0.20 and the elimination of individual items did not increase Cronbach’s alpha substantially in our Indian sample.

#### Covariates

Mothers reported their highest education levels and their child’s age. Education levels provided to participants to choose from were context dependent and were standardized using z-score. Mother were more highly educated in the U.S. and Beijing than India and Hong Kong and therefore, education levels were added to our models. Children were younger in India (mean age = 2.22 years) and the U.S. (mean age = 2.95 years) than in China (mean age = 4.77 and 4.93 years in Beijing and Hong Kong, respectively). Therefore, child’s age was included in all our models.

### Analytical plans

All analyses were conducted with standardized ES practices variables for the purpose of harmonization across our questionnaire and interview data. We addressed our first aim regarding the prevalence of maternal ES practices and socialization goals across cultural groups using two sets of MANCOVA (one for practices and one for goals), both adjusting for child age, sex, and maternal education. Univariate ANCOVA and post-hoc Tukey tests were used to identify the specific practices/goals and specific groups that showed significant differences. Partial eta squared (*ηp*^2^) were reported for effect size—0.02 indicates a small effect, 0.06 indicates a medium effect, and 0.14 indicates a large effect (Richardson, 2011).

For our second aim, we conducted a path analysis under the structural equation modeling (SEM) framework using R Lavaan (Rosseel, 2012) to investigate the association between two maternal EC goals and multiple ES responses simultaneously, again adjusting for the same covariates. Since our analysis included two EC goals as predictors that are likely to be correlated, path analysis is a well-suited approach that allows the modelling of covariances between these variables and is less prone to false positive findings—a major issue of running multiple, separate regression models.

Building on that, we used a multi-group approach in the path model to explore how these associations may be moderated by cultural groups to address our third aim. The intercepts of ES practices in the multi-group path model represent the adjusted group mean while controlling for all variables in the model, including EC goals, other ES responses, and covariates. Therefore, they are different from the group means of ES practices reported in Table 2, though both were standardized, and the estimates obtained from ANCOVA in aim 1. We fitted four progressively more restrictive model: Model 1 allowed each group to freely estimate the associations between EC goals and ES responses, Model 2 and 3 fixed the estimates of these associations to be the same across the two Chinese groups, with change in degrees of freedom (∆df) of 10, and three Asian groups (∆df = 20), respectively, and Model 4 fixed estimates for these associations across all four groups to be the same (∆df = 30). The comparisons between Model 1 and 2 tested within-Chinese differences, while the comparison between Model 1 and 3 tested within-Asian differences. A significant chi-squared difference test (*p* < 0.05) would signify that the more restricted model fitted worse than the less restricted model, indicating that the estimates are significantly different across groups and providing support for a moderating effect. Model fit was considered acceptable based on the following criteria: Comparative Fit Index (CFI) > 0.90, Root Mean Square Error Approximate (RMSEA) < 0.10, and Standardized Root Mean Squared Residual (SRMR) < 0.08 (Hoyle, 2012).

**Table 2:** Descriptive statistics of ES practices and EC goals across cultural groups.

Variables	*M*	SD
**Distress^a^**	0.00	1.00
Hong Kong	0.49	0.94
Beijing	−0.28	1.00
EA	−0.22	0.81
India	0.22	1.16
**Emotion-focused^a^**	0.00	1.00
Hong Kong	−0.20	1.07
Beijing	0.78	0.52
EA	0.03	1.03
India	−0.42	0.84
**Problem-focused^a^**	0.00	1.00
Hong Kong	−0.26	1.03
Beijing	0.59	0.88
EA	−0.32	0.79
India	0.31	1.10
**Minimizationa** ^a^	0.00	1.00
Hong Kong	0.49	0.81
Beijing	0.19	1.18
EA	−0.30	0.91
India	0.06	0.99
**Traininga** ^a^	0.00	1.00
Hong Kong	0.09	0.99
Beijing	0.54	0.65
EA	−0.29	0.94
India	0.07	1.12
**Relational EC goal**	4.87	0.64
Hong Kong	4.95	0.56
Beijing	4.93	0.68
EA	4.54	0.66
India	5.18	0.40
**Individualistic EC goal**	4.66	0.59
Hong Kong	4.55	0.43
Beijing	5.11	0.49
EA	4.41	0.62
India	4.72	0.52

^a^Standardized variable; EC goals, Emotional competence goal, EA, European Americans.

It is important to note that we decided to conduct separate sets of MANCOVA outside of the SEM framework since values of the standardized ES practices variables represent deviations from the grand mean rather than actual scores based on original measurement metric, making the interpretation of the mean differences estimated within the SEM framework less straightforward. Therefore, we first examined the mean-level group differences in ES practices separately using MANCOVA followed by ANCOVA *post hoc* tests. Then, the multi-group path models were conducted to further investigate the differential associations between EC goals and ES practices across groups.

## Results

### Preliminary analyses

Means and standard deviations of study variables are presented in Table 2. Bivariate correlations of all variables are in Supplementary Table 1C. Briefly, bivariate correlations showed that relational EC goals was significantly positively correlated with individualistic EC goals (*r* = 0.44) as well as problem-focused, distress, minimization, and training responses of increasing effect sizes (*r*s = 0.18–0.34). Individualistic EC goals was significantly negatively correlated with distress (*r* = −0.19) and positively correlated with training, problem-focused, and emotion-focused responses of increasing effect sizes (*r*s = 0.20–25). Among maternal ES behaviors, distress was positively correlated with minimization (*r* = 0.22) but negatively correlated with emotion- and problem-focused responses (*r*s = −0.27 and −0.15, respectively), while emotion- and problem-focused responses were positively correlated with each other (*r* = 0.26). Training responses were positively correlated with minimization and problem-focused responses (*r*s = 0.15 and 0.17, respectively).

### Significant differences in maternal ES practices and EC goals across cultural groups

After adjusting for child sex, age, and maternal education, there were significant differences in maternal report on ES behaviors and goals across cultural groups based on the two MANCOVAs, Wilks’ *Λ* = 0.43, *F*(20, 594.63) = 8.54, *p* < 0.001 (ES practices) and Wilks’ *Λ* = 0.65, *F*(8, 276) = 8.32, *p* < 0.001 (EC goals) (Aim 1). Follow-up univariate ANCOVA for each ES practice and goal also revealed significant differences, *F*s (3,184) = 5.46–18.46, all *p*s <0.001, with medium to large effect sizes, *ηp*^2^ = 0.08–0.23. Mothers in Hong Kong (Estimated marginal means; EMM *=* 0.60, SE *=* 0.22) reported significantly higher use of distress reactions than their counterparts in the U.S. (EMM *=* −0.20, SE *=* 0.13) and Beijing (EMM *=* −0.23, SE = 0.22). As expected, both Hong Kong and Beijing Chinese mothers reported using more minimization (HK: EMM *=* 0.92, SE *=* 0.22; Beijing: EMM *=* 0.52, SE *=* 0.22) and training responses (HK: EMM *=* 0.39, SE *=* 0.22; Beijing: EMM *=* 0.77, SE *=* 0.22) than EA mothers (minimization: EMM *=* −0.42, SE *=* 0.12; training: EMM *=* −0.39, SE *=* 0.12). In contrast, Beijing Chinese mothers reported using more emotion-focused responses (EMM *=* 1.29, SE *=* 0.20) than Hong Kong (EMM *=* 0.39, SE *=* 0.20) and EA mothers (EMM *=* −0.14, SE *=* 0.12), while both Indian (EMM *=* 0.46, SE *=* 0.17) and Beijing (EMM *=* 0.70, SE *=* 0.21) mothers reported more problem-focused responses than EA (EMM *=* −0.45, SE *=* 0.12) and Hong Kong mothers (EMM *=* −0.35, SE *=* 0.21).

There were also significant differences in maternal report on EC goals across cultural groups, *F*(3,140) = 6.43, *p* < 0.001 and *F*(3,140) = 7.13, *p* < 0.001 for individualistic and relational EC goals, respectively, based on ANCOVA after adjusting for child age, sex, and maternal education. The effect sizes, *ηp*^2^, were 0.12 and 0.13, respectively, indicating moderate effects. Post-hoc Tukey test showed that Beijing Chinese mothers (EMM = 5.04, SE *=* 0.12) reported higher endorsement of individualistic EC goals than Hong Kong (EMM = 4.52, SE *=* 0.13) and EA mothers (EMM *=* 4.41, SE *=* 0.09), *p*s = 0.002–0.004, while EA mothers (EMM *=* 4.50, SE *=* 0.10) endorsed significantly lower relational EC goals than all Asian groups, with Indian mothers rated the highest and Beijing mothers rated the lowest (EMM *=* 5.01–5.09), *p*s = 0.005–0.043.

### Path analysis indicated maternal EC goals were associated with their ES practices

**Figure 1:**
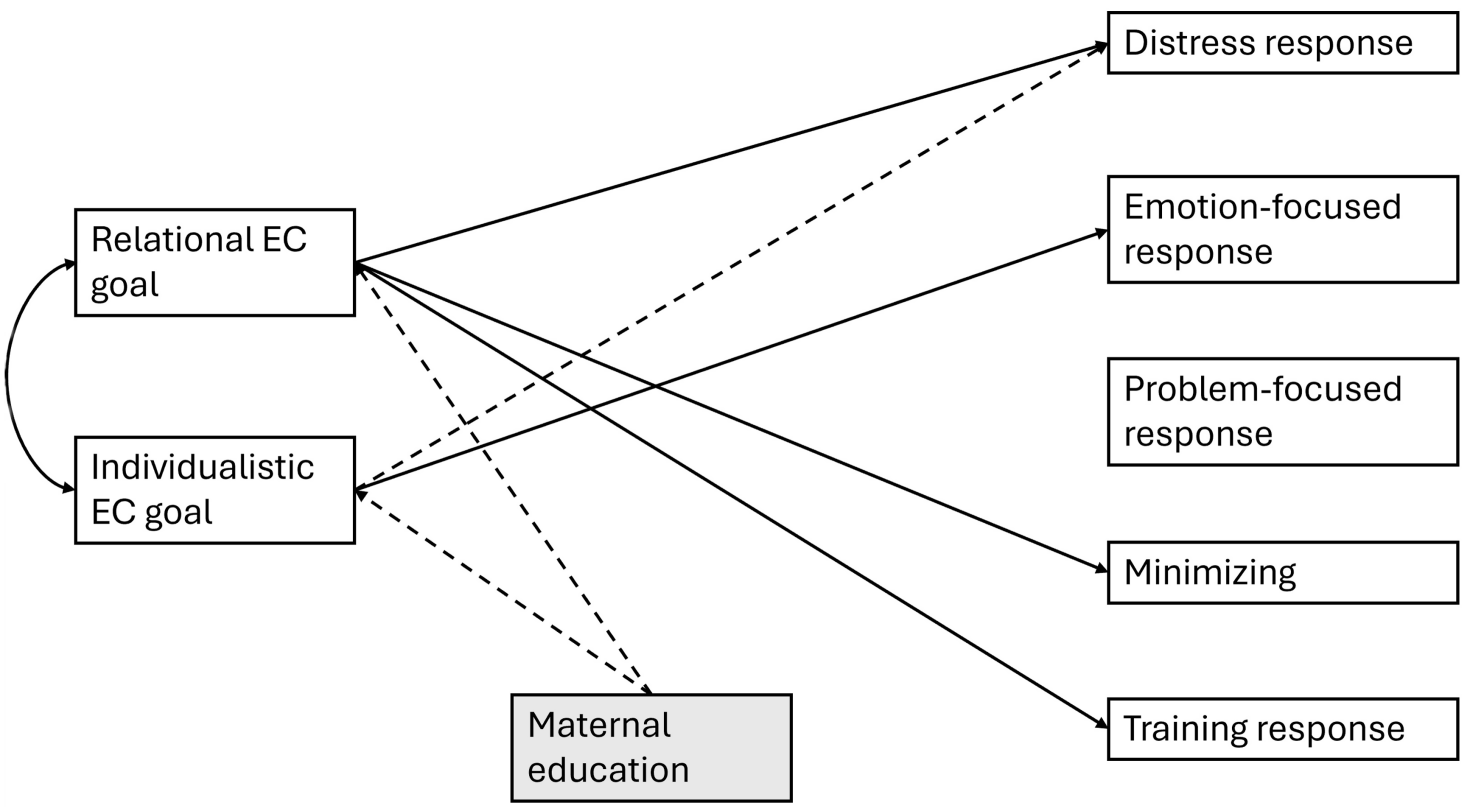
Effects of emotion competence goals on emotion socialization practices. Only significant paths (*p* < 0.05) are presented for parsimony. Solid line represents positive association and dotted line represents negative association. Child age was included in the model but was not significantly associated with any variables.

The differences in EC goals may help explain the variations in ES practices, therefore, we conducted a path analysis model to examine the overall association between EC goals and ES practices using the full sample, including all cultural groups (Aim 2; Figure 1). The model was saturated (df *=* 0); hence the model fits were all perfect. Child sex was included at first as covariate, however, since it was not significantly related to any EC practices or goals, it was removed for parsimony. Child age was kept as a covariate given its importance in ES processes and the relatively large age range in our sample. The model showed that mothers endorsed higher level of relational EC goals reported more distress, *β* = 0.31, *p* < 0.001, minimizing, *β* = 0.21, *p* = 0.020, and training responses, *β* = 0.30, *p* = 0.001, while those with higher individualistic EC goals reported less distress, *β* = −0.31, *p* < 0.001, and more emotion-focused responses, *β* = 0.19, *p* = 0.04. Thus, when the full sample was used, maternal relational and individualistic EC goals were shown to be differentially linked to their ES practices.

### Multi-group path model revealed the moderating effect of cultural group

Next, we conducted a series of multi-group path models with increasing restrictions to the parameters across groups to test the moderating effect of cultural groups and illuminate cultural-specific effects in the association between EC and particular ES behaviors (Aim 3; Table 3). In Model 1 (all paths were estimated freely across groups), only U.S. and Beijing mothers who endorsed higher relational EC goals were more likely to report distress response, *β* = 0.37, *p* = 0.013 and *β* = 0.42, *p* = 0.025, respectively, whereas this association was nonsignificant in Hong Kong and Indian samples. Beijing mothers with higher level of individualistic EC goal, but not in other cultural groups, reported less distress responses, *β* = −0.36, *p* = 0.04. Additionally, only Hong Kong mothers with higher relational EC goals reported using more emotion-focused and problem-focused responses as well as minimizing responses, *β* = 0.49, *p* = 0.03, *β* = 0.53, *p* = 0.15, and *β* = 0.44, *p* = 0.044, respectively. Indian mothers who endorsed higher level of individualistic EC goals reported using more emotion-focused responses, *β* = 0.34, *p* = 0.013. Among EA mothers, those who endorsed higher individualistic EC goals reported using more problem-focused responses, *β* = 0.42, *p* = 0.004, while endorsing higher relational EC goals was linked to more training responses, *β* = 0.42, *p* = 0.002. Children’s age was related to maternal use of training responses but with opposing directionality among India and EAs. Specifically, Indian mothers reported using more (*β* = 0.28, *p* = 0.043), but EA mothers reported using fewer (*β* = −0.27, *p* = 0.011), training responses with older children. EA mothers also used fewer emotion-focused and minimizing responses with older children, *β* = −0.50, *p* < 0.001 and *β* = −0.32, *p* = 0.004.

**Table 3:** Standardized path coefficients between relational/individualistic EC Goals and ES practices for each cultural group.

ES practice	Cultural group	Relational EC	Individualistic EC
Distress	Hong Kong	0.063	0.002
	Beijing	**0.421**	**−0.356**
	India	0.149	−0.273
	EA	**0.372**	−0.253
Emotion focused	Hong Kong	**0.492**	−0.355
	Beijing	0.004	0.119
	India	0.128	**0.335**
	EA	0.135	−0.061
Problem focused	Hong Kong	**0.533**	−0.322
	Beijing	0.176	−0.041
	India	−0.222	−0.080
	EA	−0.261	**0.422**
Minimizing	Hong Kong	**0.443**	−0.175
	Beijing	0.319	−0.109
	India	0.048	0.170
	EA	−0.145	−0.096
Training	Hong Kong	0.199	0.282
	Beijing	0.311	−0.011
	India	−0.144	−0.273
	EA	**0.421**	0.129

Bolded estimates were statistically significant (*p* < 0.05). EC, Emotional Competence goal; ES, Emotion socialization; EA, European American.

In Model 2, we constrained the path estimates of the two Chinese groups to be equal. The chi-square difference tests showed that the model fit did not significantly reduce with these additional constraints as indicated by the insignificant chi-square test differences as well as all model fit indices meeting the criteria for acceptable fit (Model 2 in Table 4). However, when estimates of all three Asian groups (Model 3) or all cultural groups (Model 4) were constrained to be the same, the model fits were worse (Table 4), indicating significant differences in path estimates across group (i.e., moderations). Full estimates for each model are included in Supplementary Table S2. These findings suggested that mothers’ EC goals had differential relations with their ES practices depending on the cultural contexts and within-cultural differences could be explained by interindividual differences in EC goals.

**Table 4:** Chi-square difference tests for moderation analyses.

Model	∆df	∆chi-square	*p*-value
1 vs. 2	10	10.98	0.34
2 vs. 3	10	23.04	0.01
2 vs. 4	20	40.46	0.004

Model 1: Completely free estimations (CFI: 1.00, RMSEA: 0.00, SRMR: 0.00); Model 2: Constraining the estimates of associations between emotion competence goals and emotion socialization behaviors to the be the same across two Chinese groups (CFI: 0.997, RMSEA: 0.045, SRMR: 0.016); Model 3: Constraining all Asian groups to be the same (CFI: 0.956, RMSEA: 0.121, SRMR: 0.048); Model 4: Constraining all cultural groups to be the same (CFI: 0.933, RMSEA: 0.122, SRMR: 0.072).

## Discussion

The current study integrated two cross-cultural studies involving four cultural groups—Hong Kong Chinese, Beijing Chinese, Indian, and EA—to address the universal and cultural-specific process involved in the emergence of cultural variations in maternal ES across and within-culture. First, we documented the cultural variations in ES practices across the four cultural groups and demonstrated that there were both similarities and differences in ES practices across Asian groups (Indian vs. Chinese) as well as within Chinese cultures. Second, we showed that emotion-related socialization goals served as a culturally embedded factor contributing to ES practices. Specifically, relational EC goals were linked to more distress responses, minimization, and training, while individualistic EC goals were related to less distress and more emotion-focused responses. However, importantly, multi-group SEM models revealed that these associations were culturally specific. The strength and challenges of harmonizing independently conducted cross-cultural studies are also discussed below.

### Nuanced variations in ES practices across and within-culture

Addressing our first aim of documenting cultural norms of maternal ES practices, our findings supported our hypothesis H1a. We showed that, when compared to EA mothers, both groups of Chinese mothers reported higher levels of minimization in response to their children’s negative emotions—typically categorized as non-supportive in Western-based studies (Friedlmeier et al., 2011). However, it is important to note that Chinese mothers also endorsed training response more often than EA mothers. Training response is a culturally-salient response grounded in the Chinese context (Chan et al., 2009b) and is more reflective of Chinese mothers’ meta-emotion philosophy that focused on directly teaching their children how to express their emotions and behave appropriately (Chan et al., 2026). Applying the broader framework of cultural model, training responses are also well-aligned with the cultural values and goal of interdependence self-construal model that focused on familial interdependence and social harmony (Friedlmeier et al., 2011; Keller et al., 2006). This adds to the existing literature of cross-cultural differences between EA and Chinese caregivers and demonstrated the importance of considering culturally-salient practices and using measures that were grounded in the cultural context (Chan, 2012; Chan et al., 2009b).

Our findings also provided evidence supporting our hypothesis H1b regarding the heterogeneity of ES practices across Asian cultures. In our sample, only Chinese mothers, but not Indian mothers, reported higher training responses than EA mothers. This finding could be explained by the differing traditional belief systems of Confucian and Hinduism across Chinese and Indian (Rao et al., 2003). Traditional Confucian beliefs emphasized the importance of parenting efforts to help overcome children’s innate tendencies through efforts and hence ES involving training is in line with such beliefs. On the contrary, traditional Hindu beliefs allow children’s innate disposition to be indulged and may not focus as much on conscious training in the early years (Saraswathi and Ganapathy, 2002). However, we also found similarities in ES practices across Chinese and Indian mothers, specifically in regard to the high endorsement of problem-focused responses. This finding is consistent with a prior study that examined ES behavioral profiles in both Indian and Chinese mothers (Trevethan et al., 2021). They found that problem-focused response is one of the highly endorsed responses, followed by explanation-oriented, which was not measured in the current study.

When we considered variations within the Chinese culture across two communities of differing historical and sociopolitical backgrounds, we continued to observe nuanced differences. Although both Chinese groups reported higher levels of training and minimization than EA mothers, Hong Kong Chinese mothers endorsed higher levels of distress, lower levels of emotion- and problem-focused responses than Beijing Chinese mothers, revealing an overall pattern of less supportive responses even comparing to their counterpart in the same culture, supporting hypothesis H1b. This finding can be corroborated by the qualitative portion of the study where we found that Hong Kong Chinese mothers felt less competent in supporting their children’s emotion regulation or learning and reported stronger concerns of shame and social judgement than Beijing mothers (Chan et al., 2026). Importantly, Beijing Chinese mothers were found to report the highest levels of emotion-focused and problem-focused responses among all cultural groups, including EA mothers. These responses were typically considered as supportive in Western-based studies. Hence, our findings challenge the traditional view that Asian parents showed lower supportive ES behaviors than EA mothers (Friedlmeier et al., 2011; Raval and Walker, 2019). Given the rapid industrialization and modernization of Chinese societies, examination on contemporary Chinese parenting practices and the underlying driving factors is warranted to avoid relying on inaccurate assumptions or empirical findings that were based on observations prior to these vast changes (Fung et al., 2017).

### Universal and cultural-specific associations between EC goals and ES practices

Parents’ socialization goals specific to emotions, such as EC goals, have been proposed to serve as culturally-embedded factors that drive the variations across and within culture (Raval and Walker, 2019). Our study made an important theoretical contribution by providing empirical evidence to support this proposed factor as driving force of cultural variations of ES both across and within culture. Our study moved away from past practices of solely relying on assumptions regarding cultural values of Asian and EA parents by directly assessing endorsement of socialization goals and delineating how these factors may drive cross-cultural variations and explain within-cultural differences. Aligned with previous studies and theories of cultural model (Chan et al., 2009b; Friedlmeier et al., 2011; Raval and Walker, 2019), mothers from all Asian groups in our samples reported higher endorsement of relational EC goals than EA mothers, supporting our hypothesis (H1c). Adding to existing literature, we found similarities in the extent to which Chinese and Indian mothers value relational EC goals.

However, there were significant cross- and within-cultural differences in endorsement of individualistic EC goals. Interestingly, Beijing mothers endorsed individualistic EC goals to the greatest degree among all four cultural groups. Although Chinese mothers were previously reported to value both relational and individualistic EC goals (Chan, 2011), we hypothesized that individualistic EC goals to be endorsed to a higher level in a supposedly independent culture such as EA culture than Chinese culture (H1c), which was not supported by our findings. Nonetheless, this follows the same shift of child-rearing beliefs and goals towards more child-directed and independence-oriented views of parenting that has been observed in Mainland Chinese parents (Chen and Chen, 2010; Fung et al., 2017). Indeed, Mainland Chinese parents nowadays were found to focus on promoting children’s autonomy, socioemotional development, and psychological well-being rather than more traditional Chinese values such as academic success and compliance (Ren and Pope Edwards, 2016; Way et al., 2013). The current findings added to the emerging evidence showing that Mainland Chinese mothers’ parenting might be increasingly individualistic and highlighted the importance of explicitly examining parental goals rather than making assumptions based on one’s cultural origins. It also emphasizes the influence of changing social context in shaping parental socialization goals (Greenfield, 2018). This social shift may work alongside with a reference-group effect, in which Beijing Chinese mothers in our sample who are more highly educated than the average Chinese mothers may identify themselves as holding more individualistic EC goals while sharing relational EC goals as core cultural goal with their “reference” (Heine et al., 2002; Noordzij et al., 2024). Therefore, future research should consider how contemporary societal shift in conjunction with reference-group effect may shape the variations in parental ES.

To address our second aim, we investigated EC goals as a potential driving factor of variations in ES practices. We found that in general, mothers’ relational EC goals contributed to more distress, minimizing, and training responses, while individualistic EC goals were associated with less distress and more emotion-focused responses. These findings aligned with our hypotheses (H2) and shed light on the universal patterns of how maternal EC goals link to their ES behaviors (Raval, 2023). Together, these associations and the observed differences in EC goals endorsement could help explain the cultural variation in ES practices. For example, Beijing mothers endorsed higher levels of individualistic EC goals and therefore they reported lower levels of distress response, as compared to other cultural groups.

However, the relevance of EC goals on ES practices may depend on cultural contexts and hence the mechanism could be culturally-specific (Rao et al., 2003; Wu et al., 2025). Using a multi-group SEM approach, we found that cultural-specific processes can be observed across EA and Asian groups as well as across Indian and Chinese mothers based on the worsened model fit when associations were restricted to be the same across these groups. This indicated that EC goals may be a stronger driving factor for ES practices in one culture than the other. However, this process did not differ significantly across Hong Kong and Beijing Chinese mothers. This finding provided insight into the level of granularity in cultural specificity, supporting the importance of testing the heterogeneity across Asian cultures but also revealing the similarities within culture.

When we estimated all associations between EC goals and ES practices freely across all cultural groups, the associations were often only significant in some specific groups but not all. For instance, relational EC goals were only linked to higher levels of training in EA mothers, but not in any Asian cultural groups. This showed that relational EC goals were a relevant factor to understanding individual differences in training responses reported by EA mothers, although this ES practice may not be as prevalent in Western culture (Fiorilli et al., 2015). Importantly, some associations were only significant in one specific group using the multi-group SEM approach but not when the universal process was examined, which is not entirely surprising given the significant moderation effect. For example, there were no significant associations between EC goals and problem-focused responses when all groups were examined together. The lack of effect observed when all cultural groups were analyzed together may be attributable to the opposing effects across cultural groups that effectively cancel one another out. Yet, in the multi-group SEM, problem-focused responses were driven by different EC goals across EA and Hong Kong mothers. While EA mothers’ problem-focused responses were guided by stronger individualistic EC goals, that of Hong Kong mothers were driven instead by stronger relational EC goals. This finding indicated that problem-focused responses may serve differential functions across cultures and thus driven by different EC goals. Triangulating the qualitative findings of this study (Chan et al., 2026), EA mothers may teach their children how to problem solve to promote their independence and self-regulation to prevent undesirable consequences of negative emotions (individualistic EC goal), while Hong Kong mothers may do so to help children overcome the emotion with the goal of avoiding such emotional reaction in the future to ensure social harmony and maintain positive social relationship (relational EC goals). Therefore, although problem-focused response is typically seen as a supportive strategy, the underlying motivation of this behavior may be ambiguous and could depend on parents’ EC goals. Future cross-cultural research should consider the cultural meaning behind the same ES practices, which may help advance our understanding on its differential effects, if any, on children’s development.

Maternal ES responses could also be influenced by child age (Holodynski and Friedlmeier, 2005; King et al., 2025), including shifting from more emotion-focused to cognitive-focused responses (Mirabile et al., 2018) and increasing expectations for children to be able to self-regulate their emotions (Stettler and Katz, 2014). Aligning with the developmental perspective on maternal ES, we also found an effect of child age on maternal ES responses and, importantly, this effect was culturally specific. Our finding of EA mothers using fewer emotion-focused responses with older children was consistent with past findings (King et al., 2025). It is important to note that our EA sample had the largest child age range, providing better opportunity to detect differences across ages. Interestingly, we also found that EA mothers of older children used fewer minimizing and training responses. This finding can potentially be explained by parents’ adjustment based on their children’s emotional competence (Mirabile et al., 2018) – older children may have better self-regulation and require fewer minimizing and training responses from their mothers. In contrast, our findings suggested that Indian mothers of older children used more training responses. Our Indian sample involved younger children, spanning across toddlerhood and early preschool-age. Therefore, it is possible that only mothers of older children in the Indian group expected their children to express emotions according to display rule and hence used training responses. Given the potential confounds between cultural groups and child age in our study, future studies with more comparable ages across cultural groups are needed to corroborate our findings of cultural-specific effects of child age.

### Strengths

Our study has important strengths that make a significant contribution to understanding how culture shape ES. First, we utilized culturally appropriate (i.e., MRCES developed in Chinese culture) and innovative measures (i.e., interview with culturally relevant vignettes) to assess ES practices that are salient in Asian contexts (e.g., training). Second, our study shed light onto cultural influence on emotion-related parenting and socialization with a focus on culturally embedded factors like socialization goals. Third, we leveraged cross-cultural data across four sites, including less represented cultural groups in the Asian context. The two Asian countries—India and China, as well as the two communities in China—Hong Kong and Beijing—allowed exploration of the often overlooked within-cultural variations. Lastly and importantly, we addressed universal and culture-specific mechanisms of how variations of ES practice emerged.

### Limitations and methodological challenges in cross-cultural study

The interpretation of the current findings should be considered with its limitations and challenges that come with harmonizing two cross-cultural studies. First, the current study leveraged data from two independent cross-cultural studies—both collected data in the U.S. with parallel data collection in one Asian country (China and India, respectively). Despite the independent data collection process, the two studies shared overarching research goals (i.e., understanding how cultural differences in ES emerged). Recognizing the limited availability of cross-cultural empirical data on ES in multiple Asian cultures, we decided to capitalize on the overlap in conceptualization and harmonize related measures in these two studies to address this research gap. However, there are important differences that should be considered when interpreting our findings. For example, the Chinese study recruited mothers with children of 3–6 years old while the Indian study recruited mothers with 1.5–3 years old children. Therefore, we adjusted for children’s age in all models to account for this sample difference. Yet, the developmental expectations and needs of toddler and preschoolers may still be different and could potentially influence mothers’ ES goals and practices depending on their cultural value (Chan et al., 2009a). Indeed, we found differential associations between children’s age and mothers’ training and emotion-focused responses in the US and India. Future studies should consider examining potential changes in mothers’ goals and behaviors across children’s developmental stages with longitudinal data. Despite age variations, the current study adds to the literature by focusing on young children (toddlers and preschoolers) while other existing studies focused on school-age children.

Second, although the total sample size was comparable to other cross-cultural ES studies (Fiorilli et al., 2015; Raval et al., 2013), the sample size of each cultural group was small, limiting the power to detect small but meaning effects, especially at the two sites in China. However, guided by the known sociocultural differences reported in past literature (Chan et al., 2026; Fung et al., 2017) and the observed differences in patterns of ES practices across the two Chinese samples in our study, we contented that the within-culture differences were important to document, therefore, we decided to keep these sites as separate groups and formally test their differences in our analyses despite the smaller sample size. Relatedly, we acknowledge that the unequal sample sizes across groups could influence our estimates in ANCOVA and path model.

Lastly, since the two studies were developed separately, different methodologies were used to assess mothers’ reactions to children’s negative emotions—questionnaire and interview in the Chinese study and India study, respectively. While the questionnaire data used Likert scales (i.e., ordinal data), the open-ended interview data were in frequency. We harmonized these data to the best of our ability by creating proportions for the variables coded from the interview then standardizing (z-score) our variables. Nonetheless, future studies using the same methodologies across all cultural sites are needed to corroborate these findings. Given that our ES practices variables were standardized across samples in our analyses, the group differences did not reflect the actual score differences, but differences in SD unit, making it less intuitive for future cross-cultural comparison that also used the well-established CCNES scales. Importantly, we chose to use culturally informed measures that have good content validity but some measures have relatively low internal reliability in specific cultural group (e.g., MRCES training subscale in Beijing and EC goals measure in India). One possible reason for the poorer reliability in our study could be that the training subscale was originally developed in samples of Chinese parents with older children (6–8 years old) and this behavior may not be consistently used by parents of younger children, like our sample, even among Chinese parents. The psychometric properties of these new, culturally grounded measures need to be established in future studies with larger sample sizes from diverse cultures. Furthermore, the cultural meanings and functions of these culturally salient ES practices still need further examination. For example, future research can extend on our current finding of high prevalence of using training responses in Chinese culture and examine how this training response is associated with child outcomes.

### Future directions

To address the limitations of the current study and advance our understanding of cultural variations in ES and children’s emotional development, future research should consider testing the associations between parental socialization goals with their ES practices over time to establish temporal precedence and across developmental stages using longitudinal designs. Given the development shift of other- and self-regulation across childhood, future studies could also compare children of varying age levels (e.g., toddlerhood, preschool-aged, school-aged) for more nuanced understanding of development. Furthermore, it is important to examine the links between parental ES practices and children’s emotional and behavioral outcomes while considering culturally embedded factors to understand the universal and specific mechanisms of children’s development across cultures. Lastly, the inclusion of fathers and other caregivers is crucial to capture the full picture of family ES as past studies have shown the unique contribution of fathers and mothers in ES (Gerhardt et al., 2020).

## Conclusion

The current study adds to the special issue collection that aims at addressing how culture shapes parenting and socialization related to children’s socioemotional development. Our findings documented the cultural variations in ES practices across EA, Chinese, and Indian cultures and illuminated on how parent’s emotion-related socialization goals—EC goals—drive their ES practices. We demonstrated the relevance of relational and individualistic EC goals in shaping mothers’ ES practices—a universal process shared across all cultural groups and unraveled the nuanced cultural-specificity in the associations between EC goals and ES practices both across and within culture. Our work has implications in early childhood education and intervention. For example, existing evidence-based parenting interventions that incorporated ES theories, such as Tuning in to Kids (Havighurst et al., 2010), should take into account parental EC goals as a potential factor of how likely parents may adopt specific ES practices based on how well the ES practices align with their EC goals. Similarly, children’s experience of socioemotional learning in classroom settings could also be shaped by teachers’ EC goals, which could vary across cultural backgrounds of teachers and further contribute to the cultural variation in ES of children.

## Data Availability

The datasets presented in this article are not readily available because the research team is conducting analyses on the data. Requests to access the datasets should be directed to chan568@purdue.edu.

## Author’s note

We offer our immense gratitude to the families in the cross-cultural study, the local collaborators (Drs. Zhenlin Wang and Li Wang) and undergraduate research assistants at each site, and the ISSBD 2 × 2 Early Career Grant that supported this work.
