# Supplementary material for: Maternal emotion socialization in Chinese, Indian, and European American families: socialization goals as a culturally embedded factor
Source: Front Psychol. 2026 Apr 23;17:1772882. doi: 10.3389/fpsyg.2026.1772882 (PMC13149158; doi:10.3389/fpsyg.2026.1772882)
Supplement: Supplementary file 1 [file Table_1.DOC]

Supplementary Material

Supplementary Table S1: Mean and standard deviation or raw scores on emotion socialization (ES) practices in Study A before standardization across samples

Supplementary Table S2: Bivariate correlations of all variables with confidence intervals

Supplementary Table S3: Multiple regressions comparing two US samples on ES practices, adjusting for child age, sex, and maternal education

Supplementary Table S4: Full path estimates of all path models (separate excel file)

Supplementary material 2: Interview material for ES practices in Study B 


Supplementary Table S1
Mean and standard deviation or raw scores on emotion socialization (ES) practices in Study A before standardization across samples

Country 	Mean	Standard deviation	
Distress response			
Hong Kong	3.29	0.78	
Beijing	2.65	0.83	
Ohio, U.S.	2.71	0.74	
Emotion-focused response			
Hong Kong	6.19	0.95	
Beijing	7.06	0.46	
Ohio, U.S.	5.81	0.69	
Problem-focused response			
Hong Kong	6.04	0.77	
Beijing	6.68	0.66	
Ohio, U.S.	5.97	0.61	
Minimizing response			
Hong Kong	4.03	0.99	
Beijing	3.66	1.45	
Ohio, U.S.	2.54	0.53	
Training response			
Hong Kong	4.57	0.67	
Beijing	4.88	0.44	
Ohio, U.S.	4.06	0.64	


Supplementary Table S2
 
Bivariate correlations of all variables with confidence intervals
 
Variable	1	2	3	4	5	6	7	8	9	
										
1. Child age	 	 	 	 	 	 	 	 	 	
 	 	 	 	 	 	 	 	 	 	
2. Maternal Education	-.02	 	 	 	 	 	 	 	 	
 	[-.16, .13]	 	 	 	 	 	 	 	 	
 	 	 	 	 	 	 	 	 	 	
3. Marital status	.18*	.24**	 	 	 	 	 	 	 	
 	[.04, .31]	[.10, .37]	 	 	 	 	 	 	 	
 	 	 	 	 	 	 	 	 	 	
4. REC	-.14	-.21**	-.41**	 	 	 	 	 	 	
 	[-.29, .03]	[-.36, -.05]	[-.53, -.26]	 	 	 	 	 	 	
 	 	 	 	 	 	 	 	 	 	
5. IEC	.10	-.27**	-.25**	.44**	 	 	 	 	 	
 	[-.06, .25]	[-.41, -.12]	[-.40, -.10]	[.31, .56]	 	 	 	 	 	
 	 	 	 	 	 	 	 	 	 	
6. Distress	-.01	-.08	-.09	.21*	-.19*	 	 	 	 	
 	[-.15, .13]	[-.22, .07]	[-.22, .06]	[.05, .35]	[-.34, -.03]	 	 	 	 	
 	 	 	 	 	 	 	 	 	 	
7. Emotion-focus	.03	-.03	-.09	.14	.25**	-.27**	 	 	 	
 	[-.11, .17]	[-.17, .12]	[-.23, .05]	[-.02, .29]	[.10, .39]	[-.40, -.13]	 	 	 	
 	 	 	 	 	 	 	 	 	 	
8. Problem-focus	.05	-.06	-.13	.18*	.23**	-.15*	.26**	 	 	
 	[-.09, .19]	[-.20, .08]	[-.26, .01]	[.02, .33]	[.07, .37]	[-.29, -.01]	[.12, .38]	 	 	
 	 	 	 	 	 	 	 	 	 	
9. Minimization	.05	-.09	-.29**	.26**	.12	.22**	.10	-.05	 	
 	[-.09, .19]	[-.22, .06]	[-.41, -.15]	[.10, .40]	[-.04, .27]	[.09, .35]	[-.04, .23]	[-.19, .09]	 	
 	 	 	 	 	 	 	 	 	 	
10. Training	.11	-.09	-.27**	.34**	.20*	.05	.13	.15*	.17*	
 	[-.04, .24]	[-.23, .05]	[-.39, -.13]	[.19, .47]	[.05, .35]	[-.09, .19]	[-.01, .27]	[.01, .28]	[.03, .31]	
 	 	 	 	 	 	 	 	 	 	

Note. M and SD are used to represent mean and standard deviation, respectively. Values in square brackets indicate the 95% confidence interval for each correlation. The confidence interval is a plausible range of population correlations that could have caused the sample correlation (Cumming, 2014). * indicates p < .05. ** indicates p < .01.

REC = Relational emotion competence; IEC = Individualistic emotion competence
Supplementary Table S3

Multiple regressions comparing two US samples on ES practices, adjusting for child age, sex, and maternal education

Variable	Estimate	SE	t	p	
Distress response					
Ohio vs. Michigan	-0.074	0.745	-0.100	0.921	
Child age	-0.166	0.104	-1.595	0.115	
Child sex	-0.195	0.178	-1.095	0.277	
Maternal education	-0.013	0.325	-0.039	0.969	
Emotion-focused					
Ohio vs. Michigan	0.220	0.823	0.267	0.790	
Child age	-0.064	0.115	-0.550	0.584	
Child sex	0.085	0.197	0.431	0.668	
Maternal education	-0.360	0.359	-1.004	0.319	
Problem-focused					
Ohio vs. Michigan	1.087	0.757	1.436	0.155	
Child age	0.173	0.106	1.634	0.107	
Child sex	-0.262	0.181	-1.444	0.153	
Maternal education	0.475	0.330	1.440	0.154	
Minimizing					
Ohio vs. Michigan	1.292	0.759	1.702	0.093	
Child age	0.025	0.106	0.238	0.813	
Child sex	0.291	0.182	1.598	0.114	
Maternal education	0.306	0.331	0.923	0.359	
Training					
Ohio vs. Michigan	0.800	0.855	0.936	0.353	
Child age	-0.119	0.120	-0.995	0.323	
Child sex	-0.010	0.205	-0.047	0.963	
Maternal education	0.093	0.373	0.250	0.803	
