# Supplementary material for: Maternal emotion socialization in Chinese, Indian, and European American families: socialization goals as a culturally embedded factor
Source: Front Psychol. 2026 Apr 23;17:1772882. doi: 10.3389/fpsyg.2026.1772882 (PMC13149158; doi:10.3389/fpsyg.2026.1772882)
Supplement: Supplementary file 3 [file Table_3.DOCX]

**Emotion Socialization Interview
About 2-Year Olds**

**Instructions for interviewer**

Use the name of the child when presenting the different situations.

**IMPORTANT: Ask follow-up questions and comments**

Some mothers may give short answers and you should ask additional questions.

Examples: The mother says: "I try to convince my child to calm down" or “I try to make my child feel better."

Follow-up questions: "Could you tell me more about how you would convince your child? Or how would you go about soothing your child?"

In some scenarios, moms would say, "I don't think my child would be upset or afraid in this situation" or they said "Something like this has not happened to me and my child before, so I don't know".

Follow-up comment: "But just imagine that you found yourself in such a situation, tell us what you think you would say and do in that situation."

I will present some situations in which XX (*name of the child*) experiences an emotion. Take a bit of time to remember or to imagine such a situation. I will ask you about your reaction in such situations.

.

1. Your child is afraid of injections and becomes quite shaky and teary while the nurse is getting his or her shot ready. How would you react? – *Give the mother some time* – How would you react? (What would you say and do?)
2. Your child gets into a disagreement with a friend or a sibling, gets angry and hits him or her. – *Give the mother some time* – How would you react? (What would you say and do?)
3. Your child just received a wonderful, very desirable toy, and reacts very happy. – *Give the mother some time* – How would you react? (What would you say and do?)
4. Your child is being left alone with a babysitter/family member and becomes quite nervous and upset when you are about to leave. – *Give the mother some time* – How would you react? (What would you say and do?)
5. Your child is participating in some group activity with his/her friends and proceeds to make a mistake and then looks embarrassed. – *Give the mother some time* – How would you react? (What would you say and do?)
6. You take away a toy from your child, because he/she was misusing it or not sharing correctly and he/she becomes angry and throws a temper tantrum. – *Give the mother some time* – How would you react? (What would you say and do?)
7. You are praising one of your children for a good mark on a school assignment. Your child becomes jealous, and calls his or her sibling a bad name. – *Give the mother some time* – How would you react? (What would you say and do?)
8. Your child is playing with a friend. The friend starts crying and your child tries to console him or her. – *Give the mother some time* – How would you react? (What would you say and do?)
9. Your child becomes sad because he/she is hurt and can’t go to the friend’s birthday party. – *Give the mother some time* – How would you react? (What would you say and do?)
10. You tell your child that his/her friend will be coming over for a visit, and he becomes visibly excited. – *Give the mother some time* – How would you react? (What would you say and do?)

Date __________________ ID: ______________

Information to the Emotion Socialization Interview

Age of son/daughter: ______ months

Gender of the son/daughter: boy girl

Time of the interview

Start: _______________

End: ________________

Additional notes to the interview:

_________________________________________________________________________________________________________________________________________________________________________________________________________________________________________________________________________________________________________________________________________________________________________________________________________________________________________________________________________________________________________
